# Benefits of applying standardized frameworks to implement psychosocial tools such as the **‘**My Logbook**’**

**DOI:** 10.1007/s00520-024-08981-7

**Published:** 2024-11-14

**Authors:** Liesa J. Weiler-Wichtl, Verena Fohn-Erhold, Verena Rosenmayr, Rita Hansl, Maximilian Hopfgartner, Katharina Pal-Handl, Verena Wasinger-Brandweiner, Kristina Herzog, Kirsten Neumann, Tobias Schellenberg, Dorothee Schönenberger-Loppacher, Christiane Faist-Schweika, Barbara Schönthaler, Mihaela Budich, Nicole Stember, Karin Wiegele, Maike Reddig, Anne Paduch, Iris Lein-Köhler, Sonja Görgen, Heike Wienands, Hiltrud Gauf, Rahel Hoffmann, Alina Kollmann, Ulrike Just, Nicole Salzmann, Petra Neunsinger, Marina Gerhardt, Stefanie Essl, Janina Borbely, Manuel Köpper, Sarah Rinner, Lisa Schubert, Ulrike Leiss

**Affiliations:** 1 Medical University of Vienna, General Hospital, Vienna, Austria; 2 Comprehensive Centre for Paediatrics Vienna, Medical University of Vienna, Vienna, Austria; 3 Department of Paediatrics and Adolescent Medicine, University Hospital Vienna, Vienna, Austria; 4 Department of Clinical Psychology and Behavioral Neuroscience, Faculty of Psychology, Technical University Dresden, Dresden, Germany; 5 Department of Psychiatry and Psychotherapy, Faculty of Medicine of the Technical University Dresden, Dresden, Germany; 6 Kinderhaus, Klinik Bad Oexen, Bad Oeynhausen, Germany; 7 Department of Paediatric Oncology/Haematology, Charité-Universitätsmedizin Berlin, Berlin, Germany; 8 Department of Paediatric Oncology and Haematology, University Hospital of Bern, Bern, Switzerland; 9 Department of Oncology/Haematology, Childhood and Adolescent Medicine, Luca-Dethlefsen-Hilfe E.V., Evangelic Clinic Bethel, Bielefeld, Germany; 10 Central Hospital Bolzano, Autonomous Province of Bolzano, Bolzano, Italy; 11 Department of Paediatric Haematology and Oncology, University Hospital Carl Gustav Carus, Technische Universität Dresden, Dresden, Germany; 12 West German Proton Therapy Center Essen (WPE), Essen, Germany; 13 Division of Paediatric Haematology and Oncology, University of Graz, Graz, Austria; 14 Paediatric Haematology and Oncology, University Children’s Hospital Muenster, Muenster, Germany; 15 Department of Paediatric Oncology and Haematology, Children’s Hospital, Hannover Medical School, Hannover, Germany; 16 Department of Paediatric Haematology and Oncology, Saarland University Medical Center, Homburg, Germany; 17 Karlsruhe City Clinic, Karlsruhe, Germany; 18 Department of Paediatrics, University Hospital Schleswig-Holstein, Kiel, Germany; 19 Children’s Hospital, Department of Paediatric Oncology and Haematology, University of Cologne, Cologne, Germany; 20 Department of Paediatric Oncology, Haematology, and Haemostaseology, Medical Faculty, University of Leipzig, Leipzig, Germany; 21 Kepler Universitätsklinikum Linz Med Campus IV, Linz, Austria; 22 Department of Haematology and Oncology, University Medical Centre Mannheim, Mannheim, Germany; 23 Cnopf’sche Kinderklinik, Nürnberg Children’s Hospital, Nuernberg, Germany; 24 Haematology and Medical Oncology, University Hospital of Regensburg, Regensburg, Germany; 25 Department of Childhood and Adolescent Medicine, University Clinic Salzburg, Salzburg, Austria; 26 Leuwaldhof, St. Veit Im Pongau, Austria; 27 Department of Childhood and Adolescent Medicine, University Clinic Tübingen, Tübingen, Germany; 28 St. Anna Children’s Hospital, Vienna, Austria; 29 Children’s Hospital, University of Würzburg, Würzburg, Germany

**Keywords:** Paediatric oncology, Quality of care, Implementation research, Feasibility, Evidence-based interventions, Psychosocial care

## Abstract

**Purpose:**

Evidence-based interventions (EBIs) are essential to improve the well-being and neurocognitive outcomes of pediatric cancer patients; however, considerable barriers hamper the implementation of these tools. The present study assessed health care professionals’ (HCP) perceived barriers and facilitators to the implementation of a specific EBI for pediatric oncology in a standardized manner to define effective solutions and practical recommendations.

**Methods:**

An adapted version of the Consolidated Framework for Implementation Research (CFIR) questionnaire was applied to inquire *n* = 31 HCPs in pediatric oncology about the five domains of implementation.

**Results:**

While most ‘*intervention characteristics*’ were considered beneficial for implementation, various aspects of the ‘*inner*’ and ‘*outer setting*’ were considered problematic. The most prevalent barriers included a *shortage in resources*, *poor integration of EBIs into policies* and *lacking incentives* such as user benefits. Concrete proposed and realized steps to facilitate effective implementation include a *patient*-*focused design* and *continuous evaluation and adaption* of the tool, a *detailed EBI user manual* and *application workshops*, as well as regular *interdisciplinary meetings* to improve communication. Regarding the internal and external settings, *involving policy makers*, *establishing psychosocial care in the insurance system* and *increasing awareness by sharing evidence* are essential steps for improved implementation.

**Conclusion:**

Based on standardized implementation evaluation, various targeted actions could be defined and implemented to facilitate successful implementation of EBIs in pediatric oncology. The results emphasize that psychosocial care must become an integral part of treatment standards and public health policies to ensure that effective psychosocial interventions for improved wellbeing and neurocognitive skills successfully reach pediatric cancer patients.

**Trial registration number:**

ClinicalTrials.gov Identifier: NCT04474678 (July 17th 2020).

**Supplementary Information:**

The online version contains supplementary material available at 10.1007/s00520-024-08981-7.

## Background

Severe and chronic illnesses such as paediatric cancer can not only cause physical symptoms and impairments but may also bring about an array of psychosocial strains to the whole family during and after acute treatment. To decrease these strains and to improve patient mental health and well-being, standards of psychosocial care have been developed, such as the standards of SIOP (Société Internationale d ‘Oncologie Pédiatrique) [1], the recommendations from the International Late Effects of Childhood Cancer Guideline Harmonization Group (IGHG) [2], the ‘Standards for Psychosocial Care for Children With Cancer and Their Families’ [3] or the PSAPOH (Psychosocial working group within the society of paediatric oncology and haematology in the German-speaking countries) guideline for psychosocial care in paediatric oncology and haematology [4]. To meet these standards, a plethora of psychosocial interventions has been developed and evaluated in clinical trials and empirical studies. Such evidence-based interventions encompass various approaches, including school reintegration, neuropsychological therapy and cognitive training for patients, counselling for parents, or group interventions for siblings to ensure that all the diverse psychosocial needs can be met [4].

Despite the evident need for and relevance of psychosocial care for paediatric cancer patients and the rigorous scientific effort to evaluate the standards and interventions, there remains a considerable gap between psychosocial research and the implementation of psychosocial care for pediatric cancer patients and survivors [5, 6]. Therefore the ‘My Logbook’ project has been developed, translating consensus- and evidence-based guidelines for psychosocial care [4] into a feasible practical guide for patient-centred care. It thereby aims to facilitate the effective application of established standards and the use of evaluated interventions. The resulting evidence-based intervention named ‘My Logbook—I know my way around’ is a multimodal tool that aims to provide standardized psychosocial care for all patients and their families throughout the entire course of disease and treatment [7]. At the same time, the tool is subdivided into ‘topic booklets’, each consisting of various methods (e.g. psychoeducation, neuropsychological interventions) to allow for the flexible adaptation of the interventions to patients’ individual needs. To circumvent possible barriers (e.g. low comparability of effectiveness due to variety of different methods) and address prerequisites of psychosocial interventions (e.g. interdisciplinarity), the patient-oriented tool is further accompanied by an expert set for health care professionals including a user manual, workshops and helpline support, regular study coordinator meetings and assessment tools. The feasibility and effectiveness of the tool ‘My Logbook’ is currently being evaluated in a multicentre study including *n* = 28 health care centres in German-speaking countries [8].

As exemplified by the described tool, adequate evidence-based interventions for pediatric cancer patients exist, yet there are several barriers to the actual implementation of EBIs, contributing to the remaining ‘care gap’ [6, 9]. For example, Morris et al. (2011) showed that the average time for an EBI to be implemented into clinical practice is 17 years [5]. To overcome this gap between research and clinical practice, the research field of ‘dissemination and implementation’ has been established [10]. Among the most prominent frameworks to promote systematic implementation, is the Consolidated Framework for Implementation Research (CFIR) [11] which has been updated recently [12, 13]. This practical framework provides tools to systematically assess 5 domains of barriers and facilitators to the effective implementation of evidence-based interventions. It helps researchers to conduct high quality evaluations while also contributing to the ‘consistent use of constructs, systematic analysis and organization of findings’ [14]. Various studies have shown that the constructs proposed by the CIFR [14] are a valuable tool for systematically and holistically investigating the complex mechanisms underlying successful implementation [15]. The insights into inner and outer settings, intervention characteristics and involvement aspects that hinder and facilitate successful implementation can then be used to effectively inform and adapt targeted interventions [16]. Despite the difficulties faced during the implementation of psychosocial EBIs, few studies have analysed barriers and facilitators in a standardized manner and the potential of frameworks has not been harnessed.

The present study uses the CFIR framework to investigate the facilitators and barriers encountered during the implementation of the tool ‘My Logbook’ in paediatric oncology in a systematic manner. To this end, a questionnaire based on the CFIR ‘Interview Guide’ was sent to an international group of multi-disciplinary health care professionals currently participating in the Quality Improvement Project—‘My Logbook!—I Know my Way Around!’. The resulting insights into the feasibility of the tool shall be used to adapt it and design actions to overcome barriers and facilitate the effective implementation.

## Methods

### Questionnaire

Following the CFIR ‘Interview Guide’ [14], an online-questionnaire was created, translated to German and adapted for the evaluation of the ‘My Logbook’ tool. After completion, the expert group surrounding the corresponding researchers evaluated the questionnaire to ensure clarity and validity. The aim of the present survey was to have an interdisciplinary, international group of German-speaking experts rate the feasibility of the ‘My Logbook’ tool regarding the different dimensions proposed by the CFIR guideline [14]. The questionnaire was distributed during the course of the study to systematically and continuously capture current information. Based on the input from regular meetings with the study coordinators at the various study sites, questions about barriers to implementations and factors supporting implementations were also included (e.g. team meetings, staff turnover, Covid-19 pandemic). All items in the CFIR were rephrased as positive statements to allow for the evaluation of the degree of agreement to the respective statement (e.g. ‘The measures for the implementation of “My Logbook” are implemented as planned.’). The response options for each item were ‘Yes’, ‘Partly’, ‘No’ and ‘Can’t judge’. Eighty percent were assigned as satisfactory level; meaning when the response ‘Can’t judge’ was removed, more than 80% of participants would have to select ‘Yes’ or ‘Partly’ combined. The detailed list of facilitators and barriers can be found in Table 2 and Table 3.

**Fig. 1:**
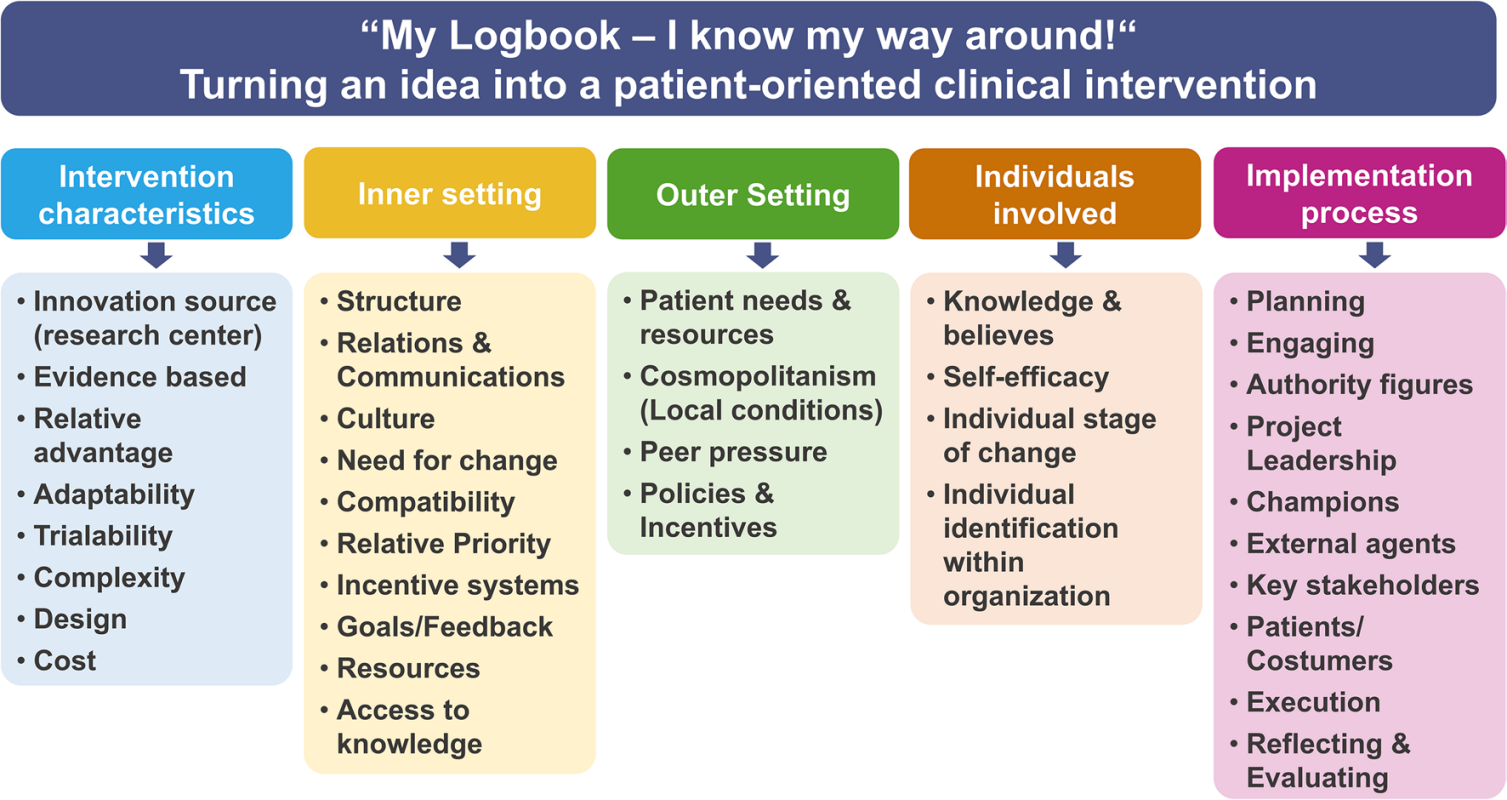
Questionnaire structure divided into the five domains and respective subcategories proposed by the CFIR-guide

The tool ‘My Logbook’ was evaluated regarding the five domains proposed by the CFIR-guide ((1) Intervention characteristics, (2) inner setting, (3) outer setting, (4) involvement and (5) implementation) and their subcategories as depicted in Fig. 1. An example for an item used would be: ‘*The tool “My Logbook” has an attractive design that is appealing to both patients and health care professionals (design, quality, packaging*).’ Also, for most domains, participants could select specific facilitators that they evaluated as useful, for example workshops or guideline-orientation to express their consensus or non-consensus. The full evaluation questionnaire (with 46 items) including an explanation for each of the assessed subcategories can be found in Supplement 1.

### Sample

Invitations to participate in the online questionnaire, which was online for two months, were sent out via email to multiple mailing lists containing participants of former delphi questionnaires [17] and members of the PSAPOH (psychosocial working group in the society for paediatric oncology and haematology in German-speaking countries). The invitation to take part in the questionnaire was sent to all professionals with a connection to the wider ‘My Logbook’ project to ensure that the participants have sufficient knowledge about the tool and its feasibility.

### Data analysis

Frequency tables and a bar plot were created for every item in the questionnaire. For better interpretation, the category ‘Can’t judge’ was removed from the answers and the relative frequency for the remaining three categories was recalculated. Based on the consensus-procedure proposed by [4], the marked level of acceptance was fixed at 80%, encompassing the answers ‘Yes’ and ‘Partly’. All results within the subdomains were merged for the analysis due to the heterogenous sample and small sample size.

Data analysis and creation of plots were conducted using the statistical software R (version 4.1.0) [18]. Data visualizations were generated using the ggplot2 package [19].

## Results

### Sample

As described in further detail in Table 1, a total of *n* = 31 participants from four German-speaking countries participated in the questionnaire which is representative of the *n* = 28 health care centres participating in the multi-national pilot of the ‘My Logbook’ tool. Most participants worked in Germany (52%) or Austria (39%). The majority were psychologists (71%) or psychotherapists (23%), while the remaining participants were either physicians, educators, nurses, art/music therapists or other. One participant reported to be a survivor of paediatric cancer. Concerning the focus of work, participants were mostly employed in acute care (68%), followed by out-patient follow-up care (32%), in-patient follow-up care (19%), rehabilitation (13%) and research (13%). Professional experience was equally distributed across the three given categories: 0 to 5 years (35%), 5 to 10 years (26%) and more than 10 years (39%).

**Table 1:** Sample characteristics (*n* = 31)

*Characteristics*	*n*	*%*
Country		
Austria	12	39
Germany	16	52
Switzerland	1	3
Italy	2	6
Occupation		
(Clinical) psychologist	22	71
Psychotherapist	7	23
Physician	2	6
Educator/pedagogue	1	3
Nurse	1	3
Art/music therapist	1	3
Survivor	1	3
Other	1	3
Focus of work		
Acute care	21	68
Out-patient follow-up care	10	32
In-patient follow-up care	6	19
Rehabilitation	4	13
Research	4	13
Other	1	3
Professional experience		
0 to 5 years	11	35
6 to 10 years	8	26
More than 10 years	12	39

### Questionnaire

**Fig. 2:**
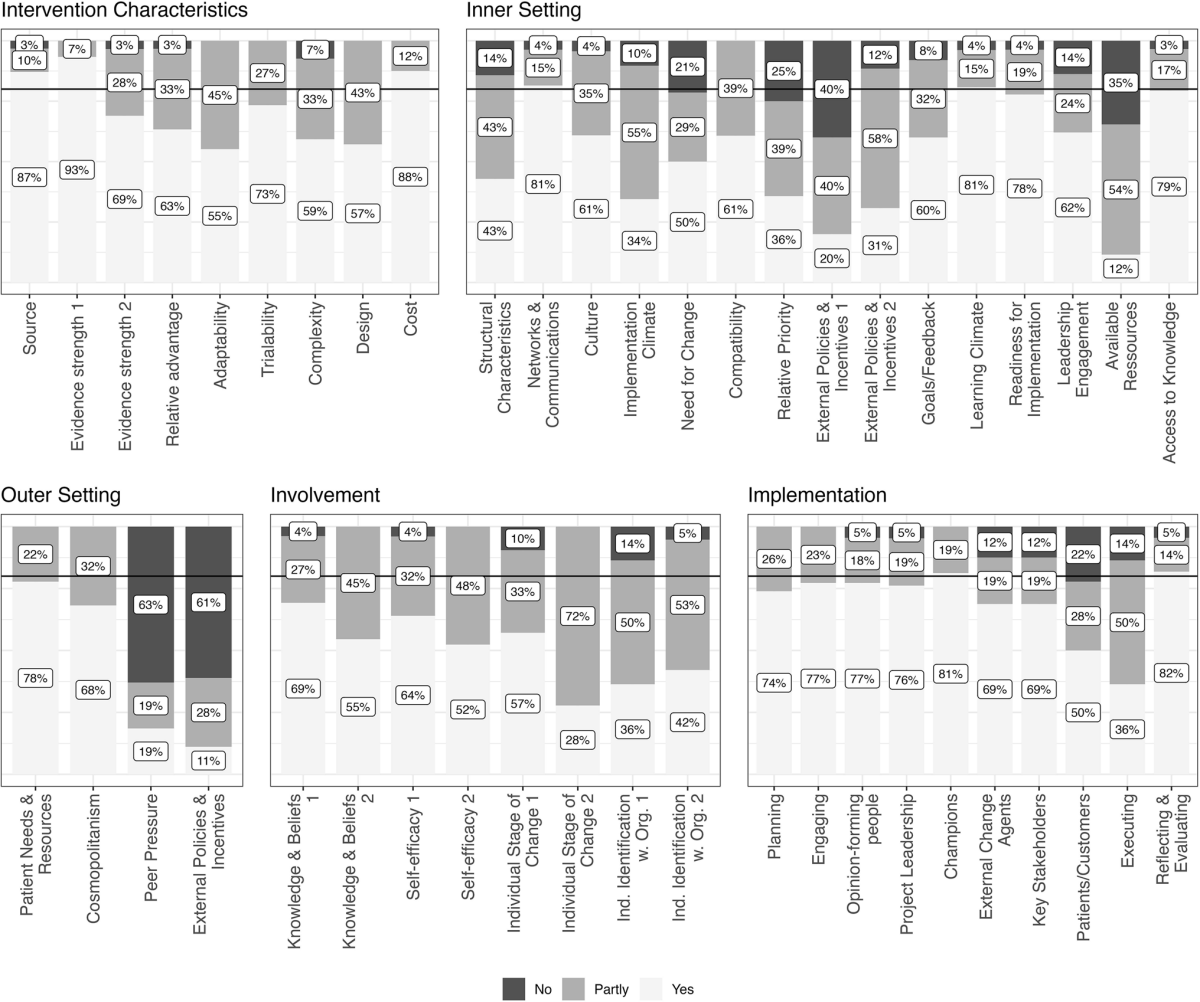
Achieved consensus for each CFIR domain and subcategory for the ‘My Logbook’ tool. The horizontal black line marks the predetermined 80% threshold for acceptance

The results provide a differentiated insight into the feasibility and implementation of the ‘My Logbook’ tool considering the CFIR domains and subcategories. The achieved consensus is illustrated in Fig. 2. The domains of ‘*intervention characteristics*’ and ‘*involvement*’ both pass the threshold of the 80% acceptance level (combined responses from ‘Yes’ and ‘partly’) in every single CFIR subcategory represented by one or more items. In total, only seven of the 46 items did not reach the acceptance level of 80%. In the following, the results are displayed for every domain ‘*intervention characteristics*’, ‘*inner setting*’, ‘*outer setting*’, ‘*involvement*’, ‘*implementation*’ within CFIR. Table 2 and Table 3 give an overview of identified facilitators and barriers in implementing the tool ‘My Logbook’ within every domain. In general, there was an increase in the number of facilitators named in relation to ‘*Intervention Characteristics*’. The facilitators are also experienced as helpful in the other categories on average, although with a lower frequency. Barriers on the other hand did not show a significant increased rate.

**Table 2:** Evaluation of facilitators relating to domains (percentage of participants considering the standard to be fulfilled)

	Intervention Characteristics	Inner Setting	Outer Setting	Implementation
	% of participants agreeing to statement			
Psychosocial expertise—In development and implementation	61	48		55
Patient-orientation—promotion of empowerment and competence to act	87**	55		48
Standards with individuality—harmonization of everyday clinical routines and maintaining diversity of methodology and individuality (objectivity)	42	32		32
Diversity of methodology and individuality (objectivity)				23
Proof of efficacy (therapy optimization study)—linking research and practice (showing that it takes expertise and time resources)	35	23		3*
Guideline-oriented—principles, objectives & measures find immediate practical application	52	19		35
Consensus-based—Interdisciplinary & International	26	13*		19
Combined tool of research and practice (connection of research and practice)	32	16		19
Making visible and strengthening the importance of psychosocial care and research (for patients and in the interdisciplinary team)	42	23		13*
The visualization of possible gaps in care (e.g. turnover in the interdisciplinary team)	39	19		26
Focus on the child as patient (age-appropriate support)	63	61		52
Concrete visualized methods (complex processes become tangible)	63	58		29
Modular structure (possibility to select specific topics)	66	58		48
Homework (transfer to everyday life as well as the possibility to work on or deepen selected tasks and exercises at home)	21	26		3*
Low-threshold contact (easier establishment of contact with a family (keyword: ‘Icebreaker’))	32	29		13*
Fixed modules create commitment (appointments can be enforced sooner, additional appointments are booked)	21	26		10*
Process-oriented screening (visibility over the course of emotional well-being and information level)	26	26		16
The flexible use by the entire PSD (Depending on the respective focus)	23	19		23
Design with high incentive character (colours, stickers, handicraft sheets, orientation aids)	47	39		29
Design oriented to the target group (font size, readability, …)	23	19		16
Design presentation (packaging, print quality, …)	18	19		6*
Trainings (workshop & training films)	68	61	52	28
Study coordination meeting (networking and exchange)	52	52	52	32
Logbook ‘Helpline’ Support (Phone & Email—(Networking and Exchange))	32	26	19	15*
Social networks (Slack, Facebook—(networking and sharing))	3*	10*	13*	8*
Conference papers, written information, etc	19	31	35	26
Newsletter	32	31	23	24
Manual (instructions as well as the sample answers in the manual support a quick use and support a practical handling)	84*	55	29	31
Basic supply with integrated ‘My Logbook’ modules	29	13*	6*	3*
Theme booklets and ‘My Logbook’	42	47	16	11*
Protocol forms for process-oriented screening (Standardized clinical assessment and psychosocial goals achieved)	19	13*	0*	3*
Evaluation aids	16	10*	6*	2*
Dashboard (presentation of scientific results)	13*	8*	10*	8*

Values with ‘*’ are below the 14th percentile, values with ‘**’ above the 84^th^

**Table 3:** Evaluation of barriers relating to domains (percentage of participants confirming the presence of barrier)

	Implementation	Inner setting
Personal assessment of the importance, significance, and topicality (of the project/topic)	26	26
Time resource	61	65
Personnel changes in the team (number of hours, sick leave, short contracts, lack of replacements during maternity/paternity leave, third-party funding…)	35	47
Restaffing during maternity/parental leave, third-party funding…)	10*	16
Study-specific organization (ethics, consent forms, data acquisition and transfer)	23	40
Scientific know-how (ethics applications, consent forms, data protection, …)	19	21
Duration and scope of the individual subject booklets	23	26
Clinic-specific framework conditions (position at the clinic, …)	29	26
Pandemic and other daily crises	23	20

Values with ‘*’ are below the 14th percentile, values with ‘**’ above the 84^th^

### Intervention characteristics

All 9 subcategories reached the acceptance level with at least 80% of participants selecting ‘Yes’ and ‘Partly’ as a response. Those subcategories included items assessing adaptability, complexity, trialability and cost, among others. Concerning the subcategory ‘*relative advantage*’ of the tool, participants highlighted the patient-orientation (80%) and psychosocial expertise (60%) as particularly useful compared to other interventions/standard care. Its consensus-orientation was only considered helpful by 26%. Concerning ‘*adaptability*’, more than 65% of participants assessed the age-appropriate materials, the visual methods and modular design of the ‘My Logbook’ tool as beneficial for implementation. Furthermore, 55% of respondents appreciated its appealing design. Regarding ‘*trialability*’, more than 80% judged the manual as useful, as did 68% for the workshops. Fifty-two percent stressed the study coordinator meetings as helpful, while different approaches were assessed as helpful by less than half of the participants. In terms of ‘*design*, *quality and packaging*’, the age-appropriate materials, the visual methods and the modular design were rated helpful by more than 60%. The aspects considered most relevant and helpful in the context of intervention characteristics were patient-orientation (87%) and as well as manuals instructing the application (84%), while dashboards and presenting scientific result (13%) and the use of social networks (3%) were rarely seen as relevant.

### Inner setting

The domain ‘*inner settings*’ consists of 15 items, eleven of which reached the satisfactory level. The subcategories ‘*networks & communication*’, ‘*compatibility*’, ‘*learning climate*’ and ‘*access to knowledge*’ were evaluated most favourably. In contrast, the item ‘*An improvement of the current situation of psychosocial care* (…) *is necessary and can be supported by* “*My Logbook*”’ was rejected by 21% of the responding professionals (Yes = 50%, Partly = 29%; subcategory: ‘*need for change*’). The subcategory ‘*relative priority*’ (‘*My Logbook*’ *has advantages compared to other methods/activities/interventions*) was evaluated as follows: Yes = 36%, Partly = 39%, No = 25%. Similarly, ‘*available resources*’ (‘*There are sufficient resources* (…) *available at my institution for the implementation of guideline-based tools such as* “*My Logbook*”’; Yes = 12%, Partly = 54%, No = 35%) and ‘*peer pressure*’ (‘*My Logbook*’ *is a* ‘*standard of care*’ *at my institutions or a fixed component of routine care*; Yes = 19%, Partly = 19%, No = 63%) did not meet the predefined level of acceptance. Regarding ‘*structural characteristics*’, 65% highlighted time resources, and around 40% each stated staff changes and study specific organization as especially challenging aspects. For ‘*networks & communication*’, more than 55% considered workshops, the manual and study coordinator meetings as helpful, while only 10% did so for social networks or the document for care standards for patients of standard risk [20, 21]. Participants assessed psychosocial expertise (48%) and patient-orientation (55%) as helpful regarding ‘*culture*’. Less than 20% each found guideline-orientation, a consensus-based approach or a combination of research and practice to be relevant. For the subcategory ‘*implementation climate*’, more than 58% of participants saw the patient-focus, the concrete visual methods of the ‘My Logbook’ tool and its modular structure as challenging. Regarding the ‘*readiness for implementation*’, workshops were seen as most helpful (61%), followed by study coordinator meetings (48%), the manual (48%) and the specific booklets (48%).

### Outer setting

The domain ‘*outer setting*’ consists of four subcategories: ‘*Patient needs & resources*’, ‘*cosmopolitanism*’, ‘*peer pressure*’ and ‘*external policies & incentives*’. Thereof, the subcategory ‘*external policies & incentives*’ (‘*Standards*, *specifications*, *political frameworks*, *recommendations or guidelines*, *pay*-*for*-*performance exist for the implementation of* ‘*My Logbook*’, *integration into the benefits catalogue of social insurance*, *cooperation or public* / *benchmark reports*’*;* Yes = 11%, Partly = 28%, No = 61%) did not reach the predefined level of acceptance. ‘*Patient needs & resources*’ and ‘*cosmopolitanism*’ reached the acceptance level without any rejections.

Concerning the subcategory ‘*cosmopolitanism*’ 52% of participants each considered study coordinator meetings and the workshops as helpful, followed by the manual (29%) and conference papers (35%).

### Involvement

The domain ‘*involvement*’, adapted from ‘*characteristics of involved individuals*’, consists of eight subcategories including ‘*knowledge & beliefs*’, ‘*self-efficacy*’ and ‘*individual stage of change*’, among others. All the positively formulated statements were perceived as adequate by over 80% of respondents.

### Implementation

The domain ‘*implementation*’ consists of ten subcategories, whereof only the item assessing ‘*patients/customers*’ was considered to be met by less than 80% of respondents (‘*There are sufficiently effective strategies to attract and involve patients in the implementation or use of* ‘*My Logbook*’ (*e*.*g*. *reports in parent association magazines*, *flyers*, *information through the psychosocial service*, *newsletters*); Yes = 50%, Partly = 28%, No = 22%). The subcategories considered most favourable for implementation were ‘*reflecting & evaluating*’, ‘*champions*’ and ‘*engaging*’.

Concerning the subcategory ‘*planning*’, 55% assessed psychosocial expertise as helpful, 48% the patient-orientation and each over 30% the guideline-orientation and having standard with a degree of individuality. In contrast, ‘*making visible and strengthening the importance of psychosocial care and research*’ and a ‘*proof of efficacy*’ was only considered relevant by 13% and 3% respectively. Regarding the subcategory ‘*engaging*’, the patient-orientation (52%) and modularity (48%) and the manual (42%) of the ‘My Logbook’ booklets were considered helpful. Social networks, homework and packaging were selected by less than 6% of participants. Barriers that were frequently chosen for the subcategory ‘*execution*’ were time resources (61%), staff changes (35%) and clinic-specific circumstances (29%). For ‘*reflecting & evaluating*’, participants evaluated the study coordinator meetings (48%) and the newsletter (42%) as useful. Also, the workshops (26%) and conference papers (29%) were often selected.

## Discussion

In this study, an international group of multi-disciplinary health care professionals was asked to evaluate the feasibility of implementing the tool ‘My Logbook’ regarding the five domains: *intervention characteristics*, *inner setting*, *outer setting*, *involvement* and *implementation*. To ensure the systematic and holistic evaluation of each domain, a questionnaire proposed by the CFIR-framework [12] was adapted to German language and professionals had to either assent to or reject positively formulated statements for each subcategory. The repeated survey served as a dynamic tool to collect insights from participants, allowing for real-time adjustments and adaptations to the ongoing implementation of the ‘My Logbook’ intervention. This approach facilitated a continuous and responsive data collection process, ensuring a comprehensive understanding of the implementation challenges and facilitators throughout the study.

Most of the given items reached the acceptance level, with the *patient*-*orientated* design and the provided *user manuals* being the features rated most beneficial for implementation. In contrast, 79% of respondents stressed a *need for change in the psychosocial care system*, and less than 80% of the inquired health care professionals found the current *availability of resources* and *external policies & incentives* to be beneficial for the implementation of the psychosocial care tool. Most respondents found that the ‘My Logbook’ tool met all the required aspects of patient *involvement*. Regarding *implementation*, the *strategies to involve patients* were not considered satisfactory by all participants, while continuous *reflection & evaluation* were considered most favourable. Therefore, the characteristics considered most beneficial for the implementation of the tool ‘My Logbook’ were predominantly related to the intervention itself, while most barriers were situated in the *outer setting*. Although the findings are specific to the tool ‘My Logbook’, it can be assumed that many of the aspects are generalizable to other fields and are therefore useful and applicable in different programmes.

**Fig. 3:**
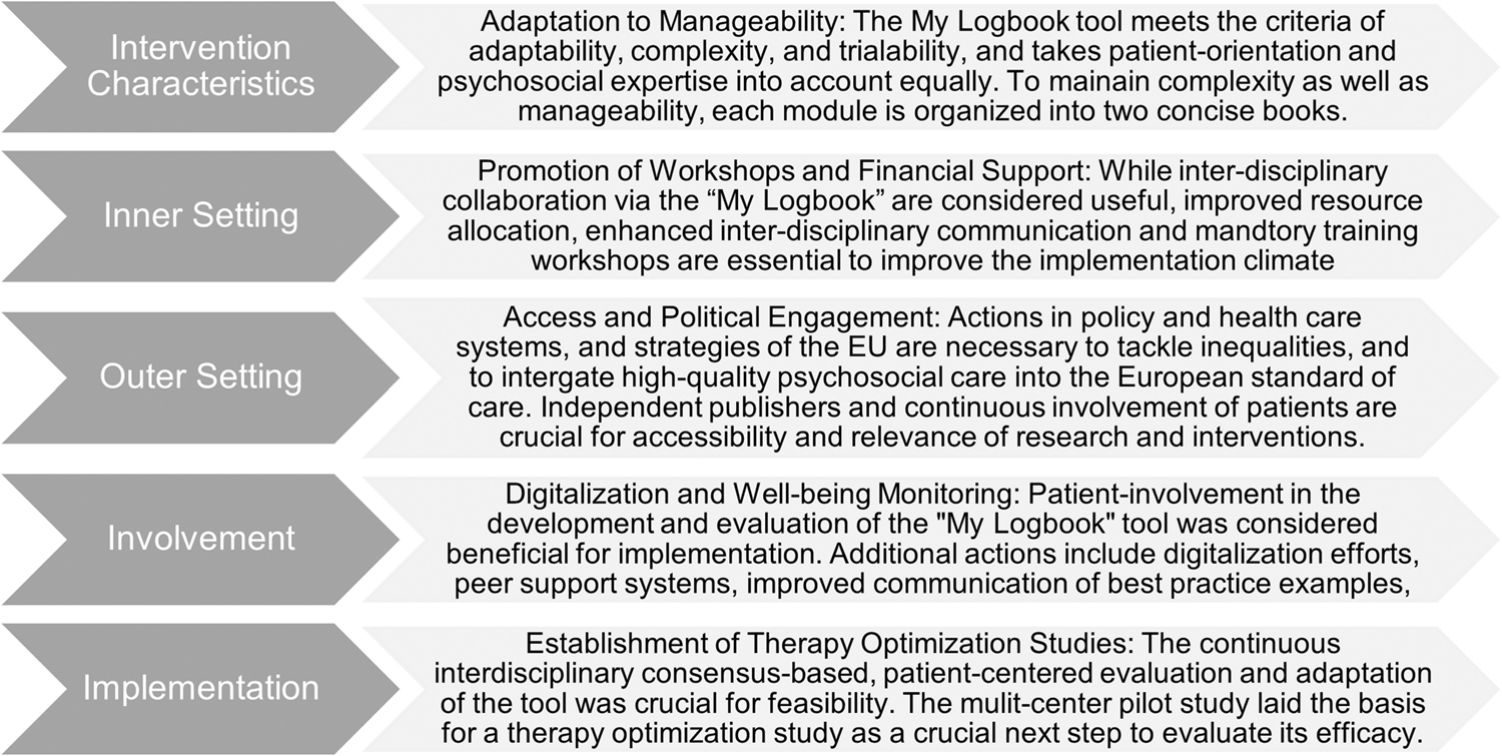
Info box: key findings per CFIR domain

Since the efficacy and relevance of psychosocial EBIs has been shown in a myriad of studies, it is now time to find and evaluate methods for the effective implementation of such tools. Using the CFIR framework to systematically inquire practicing professionals [14], the present study revealed novel and relevant insights into differentiated facilitators and barriers faced during the implementation of a specific EBIs for standardized psychosocial care in the field of paediatric. Based on these insights, targeted actions could be developed, and recommendations formulated to effectively facilitate successful implementation. Based on the feedback, detailed user-manual and training workshops were developed to facilitate the correct and standardized application of the tool. Moreover, regular interdisciplinary meetings and communicative workshops are scheduled to improve the consideration of the tool and to continuously adapt the EBI to multi-faceted views of all interdisciplinary stakeholders as well as patients. This shall allow for the tool to be accepted by professionals in different domains and to effectively target patients’ individual needs. Furthermore, the results highlight the need for structural and policy changes, including revised resource allocation and the recruitment of sufficient qualified psychosocial staff to allow for EBIs to be implemented in clinical practice, thereby ensuring and improving quality of care. Similar to pharmaceutical or medical studies, psychosocial EBIs should also be tested in therapy-optimization-studies to ensure quality of care, and comparability of care in all centres. Figure 3 provides an overview of the key findings and proposed actions to overcome the identified barriers divided per domain.

One characteristic that was considered especially beneficial for the implementation of the ‘My Logbook’ tool was the patient-orientation of the tool. This stresses the relevance of this key aim of the tool ‘My Logbook’, namely the empowerment and increased competence of patients to understand their diagnosis and to be able to shape and influence their own treatment trajectory. Additional benefits were seen in the user manuals with detailed instructions on the administration of the tool, to ensure its adequate application. Specific barriers identified by the psychosocial health care professionals mainly concerned the inner and outer setting. In the clinics, a lack of priority and incentives as well as limited resources are among the most prevalent barriers. Moreover, in the inner setting, the patient-focus and modular structure of the ‘My Logbook’ was perceived as difficult. Since these two features are crucial for individualized, patient-centred care, the reduced appreciation most likely reflects that in German-speaking countries such individualized and patient-centred care is not common and that professionals need more training to be able to apply these tools adequately. Concerning the outer setting, the health care professionals considered a lack of standardization and translation into policy and social security systems to be the major barrier. In line with findings by authors such as Wiener et al. (2020), it therefore becomes evident that despite a high quality and benefit of the tool itself, there is a clear need for the integration of psychosocial care into policies and standard-of-care procedures to ensure that EBIs become feasible for the use in clinical practice. Particularly incentives such as benefits for users and sufficient resources are necessary to ensure that not only the best established and cost-effective but the most effective and efficient interventions are used to provide the best possible quality of care to patients. Interestingly, proofs of the efficacy of new EBIs as well as the visibility of the relevance of psychosocial care were barely rated as relevant contributors to the implementation of the ‘My Logbook’ tool. This further indicates the need for EBIs and lobby work to become a more integral part of the psychosocial landscape. Moreover, close collaboration with publishers and continuously sharing benefits of psychosocial EBIs in the scientific [22, 23] as well as the patient community are important steps towards the improved awareness for their relevance and as well as better availability and user friendliness.

Overall, the results show that rather than constantly developing new interventions, it is necessary to ensure that the EBIs provided by psychosocial research can effectively make their way into clinical practice. Actual care systems in German-speaking countries (D-A-Ch region) show already a high level of psychosocial care. Unlikely, daily clinical care faces manifold barriers which lead to unnecessary gaps between evidence and clinical practice [6]. Considering the increasing amount of evidence showing the barriers of implementation and to further improve quality and standards of psychosocial care in paediatric oncology, it is overdue to act upon the evident deficits by actively trying to bridge this gap [6, 12]. Therefore, specific and mandatory standards of care such as the guideline for psychosocial care in paediatric oncology by Schröder et al. (2019) as well as more resources to implement patient-oriented care are necessary to facilitate a sustainable integration of psychosocial care into clinical practice. While the low sample size and the restriction to German speaking countries are limitations to the generalizability of the results, the selection of study cites working in accordance with the same standards of care ensured homogeneity. The present study as part of the multi-centre pilot evaluation can hence not only be viewed as a best practice example for improving the feasibility and implementation of newly developed EBIs, the pilot study also served as a crucial step towards a randomized therapy optimization study.

## Implications for behavioral health

To ensure the efficacy and feasibility of newly developed psychosocial interventions, it is crucial to evaluate the feasibility of their implementation into clinical practice. The application of tools provided by the CFIR allowed for the systematic revealed barriers and facilitators to the implementation of the patient-oriented psychosocial therapy tool ‘My Logbook’ as perceived by practicing professionals. The results showed that despite a high estimation of the patient-oriented benefits and effectiveness of the presently evaluated tool, there are various policy- and resource-related aspects of the inner and outer setting leading to a poor evaluation of the feasibility of the intervention by health care professionals. Hence there is a need for better integration of psychosocial EBIs into the standards of care in paediatric oncology—also involving political actions—to close that gap between research and practice and to ensure that pediatric cancer patients receive the best care possible and available. Based on these findings, initiatives and additional resources such as open-source communication of findings, user training workshops, a detailed user manual and continuous interdisciplinary evaluation meetings have been established to further facilitate implementation. Moreover, recommendations for more beneficial inner and outer settings were formulated targeting policy makers, and psychosocial care infrastructure to facilitate a better general integration of psychosocial care into the health care system.

Beyond the systematic evaluation and improvement of the assessed ‘My Logbook’ tool, the present study represents a concrete use-case and a crucial step towards tailoring implementation research of psychosocial tools by translating the CFIR framework into a questionnaire in German language. This not only ensures the relevance of findings in the local context but also sets the stage for future research endeavors in the German-speaking healthcare landscape. The creation of a German questionnaire provides a valuable resource for further investigations into the implementation of psychosocial tools, fostering a deeper understanding of the unique challenges and facilitators within this specific cultural and linguistic setting. This pioneering effort emphasizes the importance of continued research in adapting implementation science methodologies to diverse healthcare environments.

## Supplementary Information

Below is the link to the electronic supplementary material.

Supplementary file1 CFIR-based online survey questionnaire. (PDF 1.39 MB)

Supplementary file2 Data online survey. (XLSX 129 KB)

## Data Availability

The datasets generated and/or analysed during the current study are available from the corresponding author on reasonable request.

## References

[1] Kowalczyk JR, Samardakiewicz M, Fitzgerald E, Essiaf S, Ladenstein R, Vassal G, Kienesberger A, Pritchard-Jones K (2014) Towards reducing inequalities: european standards of care for children with cancer. Eur J Cancer 50:481–485. 10.1016/j.ejca.2013.11.004 10.1016/j.ejca.2013.11.004 24300454

[2] Marchak JG, Christen S, Mulder RL et al (2022) Recommendations for the surveillance of mental health problems in childhood, adolescent, and young adult cancer survivors: a report from the International Late Effects of Childhood Cancer Guideline Harmonization Group. Lancet Oncol 23:e184–e196 35358467 10.1016/S1470-2045(21)00750-6 PMC9639707

[3] Wiener L, Kazak AE, Noll RB et al (2015) Standards for the psychosocial care of children with cancer and their families: an introduction to the special issue. Pediatr Blood Cancer 62:419–424. 10.1002/pbc 26397836 10.1002/pbc.25675 PMC6397048

[4] Schröder HM, Lilienthal S, Schreiber-Gollwitzer BM, Grießmeier B, Hesselbarth B, Lein-Köhler I, Nest A, Weiler-Wichtl LJ, Leiss U (2019) Psychosoziale versorgung in der pädiatrischen onkologie. In: Psychosoziale Arbeitsgemeinschaft in der Gesellschaft für Pädiatrische Onkologie und Hämatologie (PSAPOH) (Issue AWMF-Leitlinie Register Nr. 025/002). 10.1007/s00761-016-0089-2

[5] Morris ZS, Wooding S, Grant J (2011) The answer is 17 years, what is the question: understanding time lags in translational research. J R Soc Med 104:510–520. 10.1258/jrsm.2011.110180 22179294 10.1258/jrsm.2011.110180 PMC3241518

[6] Wiener L, Canter K, Long K et al (2020) Pediatric Psychosocial Standards of Care in action: Research that bridges the gap from need to implementation. Psychooncology 29:2033–2040. 10.1002/pon.5505 32748495 10.1002/pon.5505 PMC8447234

[7] Weiler-Wichtl LJ, Fohn-Erhold V, Schneider C, Schwarzinger A, Krottendorfer K, Pletschko T, Rosenmayr V, Gojo J, Peyrl A, Dieckmann K, Kollmann AS, Hansl R, Slavc I, Fries J, Hopfgartner M, Leiss U (2023) Bridging the gap: A quality improvement project to implement psychosocial care standards into clinical practice in pediatric oncology. Klin Padiatr 235:350–359. 10.1055/a-2104-1049 10.1055/a-2104-1049 PMC10635755 37494589

[8] Weiler-Wichtl LJ (2020) Quality improvement project - “My Logbook! - I Know my Way Around!”; (“Mein Logbuch - Ich Kenne Mich Aus!”). In: ClinicalTrials.gov. https://clinicaltrials.gov/ct2/show/NCT04474678. Accessed 11 Nov 2024

[9] Pereira VC, Silva SN, Carvalho VKS et al (2022) Strategies for the implementation of clinical practice guidelines in public health: an overview of systematic reviews. Health Res Policy Syst 20:13. 10.1186/s12961-022-00815-4 35073897 10.1186/s12961-022-00815-4 PMC8785489

[10] McHugh RK, Barlow DH (2012) Dissemination and implementation of evidence-based psychological treatments. Oxford University Press Inc, Oxford, UK 10.1037/a0018121 20141263

[11] Kirk MA, Kelley C, Yankey N et al (2016) A systematic review of the use of the consolidated framework for implementation research. Implement Sci 11. 10.1186/s13012-016-0437-z 10.1186/s13012-016-0437-z PMC4869309 27189233

[12] Damschroder LJ, Reardon CM, Widerquist MAO, Lowery J (2022) The updated consolidated framework for implementation research based on user feedback. Implement Sci 17:1–16. 10.1186/s13012-022-01245-0 36309746 10.1186/s13012-022-01245-0 PMC9617234

[13] Damschroder LJ, Aron DC, Keith RE et al (2009) Fostering implementation of health services research findings into practice: a consolidated framework for advancing implementation science. Implement Sci 4:50. 10.1186/1748-5908-4-50 19664226 10.1186/1748-5908-4-50 PMC2736161

[14] CFIR Research Team-Center for Clinical Management Research (2023) The consolidated framework for implementation research (CFIR). https://cfirguide.org/. Accessed 6 Mar 2023

[15] King DK, Shoup JA, Raebel MA et al (2020) Planning for implementation success using RE-AIM and CFIR frameworks: a qualitative study. Front Public Health 8:1–14. 10.3389/fpubh.2020.00059 32195217 10.3389/fpubh.2020.00059 PMC7063029

[16] Breimaier HE, Heckemann B, Halfens RJG, Lohrmann C (2015) The consolidated framework for implementation research (CFIR): a useful theoretical framework for guiding and evaluating a guideline implementation process in a hospital-based nursing practice. BMC Nurs 14:1–9. 10.1186/s12912-015-0088-4 26269693 10.1186/s12912-015-0088-4 PMC4533946

[17] Weiler-Wichtl LJ, Kollmann AS, Fohn-Erhold V, Schneider C, Rosenmayr V, Hansl R, Hopfgartner M, Fries J, Herzog K, Leiss U (2023) The oracle of D-A-Ch - Results of a Delphi Survey for the development of the evidence- and consensus-based tool "My Logbook". Klin Padiatr. 10.1055/a-2135-4337 10.1055/a-2135-4337 PMC12245515 38049103

[18] R Core Team (2021) R: a language and environment for statistical computing. In: R Foundation for Statistical Computing. https://www.r-project.org/. Accessed 11 Nov 2024

[19] Wickham H (2016) ggplot2: Elegant graphics for data analysis second edition (vol. 35, 2 edn). Springer Nature. http://www.springer.com/series/6991

[20] Kazak AE (2006) Pediatric Psychosocial Preventative Health Model (PPPHM): Research, practice, and collaboration in pediatric family systems medicine. Fam Syst Health 24:381–395. 10.1037/1091-7527.24.4.381

[21] Nest A, Schreiber-Gollwitzer BM, Hesselbarth B et al (2022) Psychosoziale Basisversorgung in der pädiatrischen Onkologie und Hämatologie Version 2.0 GPOH. https://www.gpoh.de/kinderkrebsinfo/content/e1676/e176475/e176588/e258368/PsychosozialeBasisversorgung_2.0_2022-08-30_ger.pdf. Accessed 11 Nov 2024

[22] Weiler-Wichtl L, Fohn-Erhold V, Kollmann A et al (2023) Abstract O152/#1669 CCI: providing psychosocial support 01. A psychological therapy program “My logbook – my way around” to navigate children becoming experts of their own health and disease. In: Abstracts. Pediatr Blood Cancer, 70:e30748. 10.1002/pbc.30748

[23] Weiler-Wichtl L, Fohn-Erhold V, Kollmann A et al (2022) EP597 / #1656 Results of feasibility—what does it take to implement standardized psychosocial tools: “MEIN LOGBUCH – ICH KENNE MICH AUS!” („MY LOGBOOK“). In: Abstracts from the 54th Congress of the International Society of Paediatric Oncology (SIOP) e29952

[24] Ogrinc G, Davies L, Goodman D et al (2016) SQUIRE 2.0 (Standards for quality improvement reporting excellence): Revised publication guidelines from a detailed consensus process. J Nurs Care Qual 31:1–8 26429125 10.1097/NCQ.0000000000000153 PMC5411027

