# Supplementary material for: Benefits of applying standardized frameworks to implement psychosocial tools such as the ‘My Logbook’
Source: Support Care Cancer. 2024 Nov 14;32(12):789. doi: 10.1007/s00520-024-08981-7 (PMC11561021; doi:10.1007/s00520-024-08981-7)
Supplement: Supplementary file 1 — Supplementary file1 CFIR-based online survey questionnaire. (PDF 1.39 MB) [file 520_2024_8981_MOESM1_ESM.pdf]

"My Logbook" - a psychosocial tool incorporating health care professionals' perspective via a Consolidated Framework for Implementation Research (CFIR)

Liesa J. Weiler-Wichtl[1], Verena Fohn-Erhold[1], Verena Rosenmayr[1], Rita Hansl[1], Maximilian Hopfgartner[1], and Ulrike Leiss[1]

[1] Medical University of Vienna, General Hospital, Vienna Austria

Supplemental material 1. CFIR-based online survey questionnaire

Page 1

Welcome to the survey "My logbook - I know my way around!" That´s how it works!

Privacy policy

As part of the survey conducted as part of the project "My Logbook - I know my way around!", the Medical University of Vienna (MedUni Vienna), as the data protection officer, will evaluate your answers you provide.

In doing so, the company SoSci Survey GmbH, (Marianne-Brandt-Str. 29, 80807 Munich, Germany) with which MedUni Vienna has concluded an agreement on commissioned processing pursuant to Article 28 DSGVO, to provide you with the online questionnaire. The privacy policy of SoSci Survey can be found here: https://www.soscisurvey.de/de/dataprotection. The company headquarters as well as the server are located in Germany, so the strict data protection requirements of the General Data Protection Regulation also apply to SoSci Survey GmbH. Within the scope of this survey, personal data about you will be collected and processed.

A fundamental distinction must be made between:

1. those personal data by means of which a person is directly identifiable (e.g. name, date of birth, address, social security number, picture recordings...)
2. pseudonymized personal data, i.e. data in which all information that allows direct conclusions to be drawn about the specific person is either removed, replaced by a code (e.g. a number) or (e.g. in the case of image recordings) made unrecognizable. However, despite compliance with these measures, it cannot be completely ruled out that re-identification will occur.
3. anonymized data, for which a traceability to the specific person can be ruled out. Within the scope of this survey, we process data such as gender, age group, nationality, mother tongue, occupational group, diagnosis group on the basis of your consent, which can be revoked at any time, and which you express by completing and submitting the online survey expressed by filling out and submitting the online survey.

In the context of this questionnaire survey, no transfer of data outside the EU or EEA (third country) is intended.

Your consent forms the legal basis for the processing of your personal data. You can revoke your consent at any time without giving reasons. The revocation of the consent does not affect the lawfulness of the processing carried out on the basis of the consent until the revocation.

The data disclosed by filling out the questionnaire will be stored for a maximum period of 10 years from the end of the project exclusively on a password-protected workstation of the MedUni Vienna.

In principle, you have the rights to information, correction, deletion, restriction of processing, data portability and objection. To exercise your rights, please contact to liesa.weiler@meduniwien.ac.at and/or to the MedUni Vienna Data Protection Officer at datenschutz@meduniwien.ac.at.

If you are of the opinion that the processing of your data violates data protection law or that your data protection rights have otherwise been violated in any way, you may lodge a complaint with the supervisory authority (https://www.dsb.gv.at/).

Declaration of consent
• I have read and acknowledged the privacy policy.

Page 2

Dear colleagues! Dear Logbook Experts! Dear people interested in the logbook!

As many of you already know, we have been working since 2015 in close cooperation with the PSAPOH and numerous colleagues on the Project "My Logbook - I know my way around!" (My Logbook (MedUni Projects). The logbook is a guide from diagnosis to aftercare directly for young patients. directly for young patients (currently between 6 and 14 years), but it is also a direct translation of our S3-guideline (S3-guideline). guideline (S3-Guideline Psychosocial Care in Pediatric Oncology and Hematology) on psychosocial care. In "My Logbook" psychosocial processes are visualized and thus standardized. The process-accompanying evaluation for standardization of clinical assessment enables us to take a differentiated look at how the emotional well-being and the level of knowledge of the young patients - who can thus become experts in their own health (clinical trials). Trials). "My Logbook" thus bridges the gap between care and research.

Pandemics, scarce resources, staff changes and general conditions push us as psychosocial staff to our limits. limits. But that is exactly why we are convinced that standardized tools are needed to clarify what we do on a daily basis and what it takes to achieve this. and what it takes for us to provide good, quality care. In the course of our joint my logbook", we have already been able to gather valuable feedback from numerous colleagues on the obstacles that need to be overcome and the and, at the same time, what is needed for treatment tools such as "My Logbook" to reach the clinics of care/directly to the patients. with the patients.

We have summarized all of this and packaged it into a framework model (CFIR Guide):

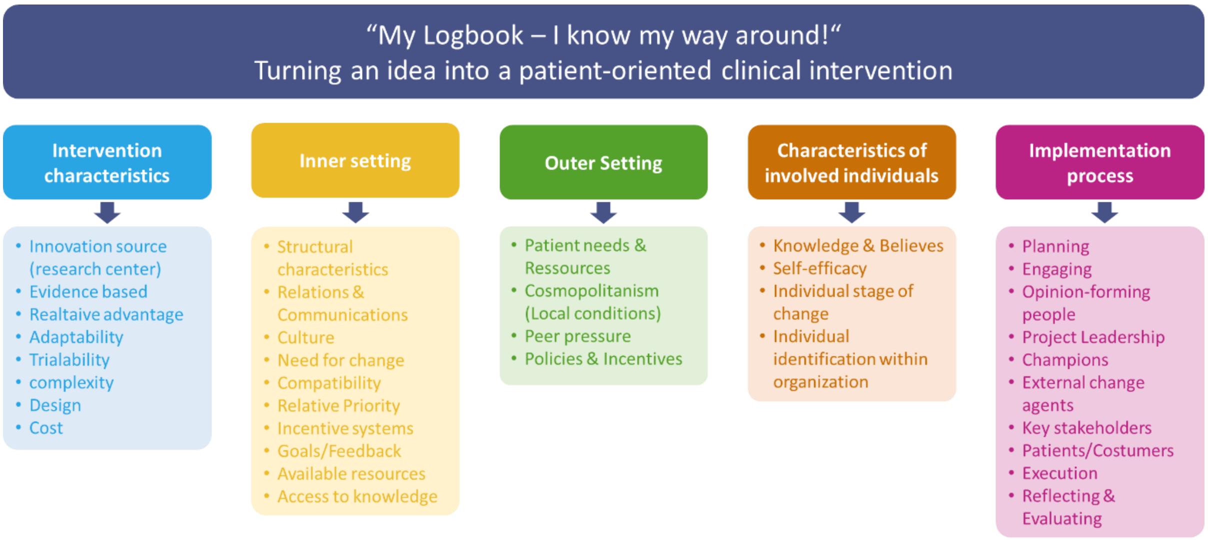

This has now resulted in a structured questionnaire, so that we can systematically identify potential opportunities and obstacles and derive concrete measures so that "My Logbook" can be applied and become a permanent building block within psychosocial care and research.

The survey should take about 30 min to complete. As usual, it will be possible to cache this time as well. Please send your feedback until 30.8.2022

Many thanks to you,

Liesa, Verena and Max

Page 3

**Place of work/institution:**
Please select the appropriate country.
• Austria
• Germany
• Switzerland
• Italy
• Other:

**Occupation:**
Please select as many occupations as applicable.
• (Clinical) Psychologist
• Educator/Pedagogue
• Social worker
• Social pedagogue
• Art therapist/music therapist
• Psychotherapist
• Physician
• Health and medical nurse
• Functional therapist
• Medical assistant
• Survivor
• Patient Advocate
• Other:

**Work Experience:**
How long have you worked professionally in the field of oncology and hematology?
• 0-5 years
• 6-10 years
• more than 10 years

**Focus of activity:**
Please select which area you primarily work in:
• Acute care
• Inpatient aftercare
• Outpatient aftercare
• Rehabilitation
• Science
• Other:

**Employment extent**
My extent of employment in the field of oncology/hematology:
____ (hours/week)
Proportion of third-party funding: ___ %

**Are you also scientifically active?**
• Yes
• No

Page 4

Is this also included in the job description?
Yes
No

Are there people on your team who are employed specifically for psychosocial research?
Yes
No

To what extent are the persons employed for psychosocial research?
Permanent employment
Third-party funding
Project-related

I was already involved in "My Logbook" in this way:
Study Center
Expert:inside group (Developed topic booklets)
Delphiexpert
Workshop
Conference - Information
Newsletter
Other
I have not yet had the opportunity

My association with "My Logbook", My personal highlight:
(open response)

Who uses the logbook at your study center?
Only I use the logbook
I and other people from my team use the logbook

How many people from your team use the logbook?
(open response)

What other professional group(s) use the logbook?
(Clinical) Psychologist
Educator/Pedagogue
Social worker
Social pedagogue
Art therapist/music therapist
Psychotherapist
Physician
Health and medical nurse
Functional therapist
Medical assistant
Survivor
Patient Advocate
Other:

When did you start using "My Logbook"?
_______ (months/year) (optional)

Which study center is it?
_______ (optional)

Page 5

Characteristics of the intervention

In the assessment of the characteristics of the intervention, the focus is on the patient-oriented tool "My logbook - I know my way around!". The key characteristics of the tool are addressed, and thus include the personal logbook with the specific topic booklets, the process-accompanying evaluation as well as the experts (manual etc.)

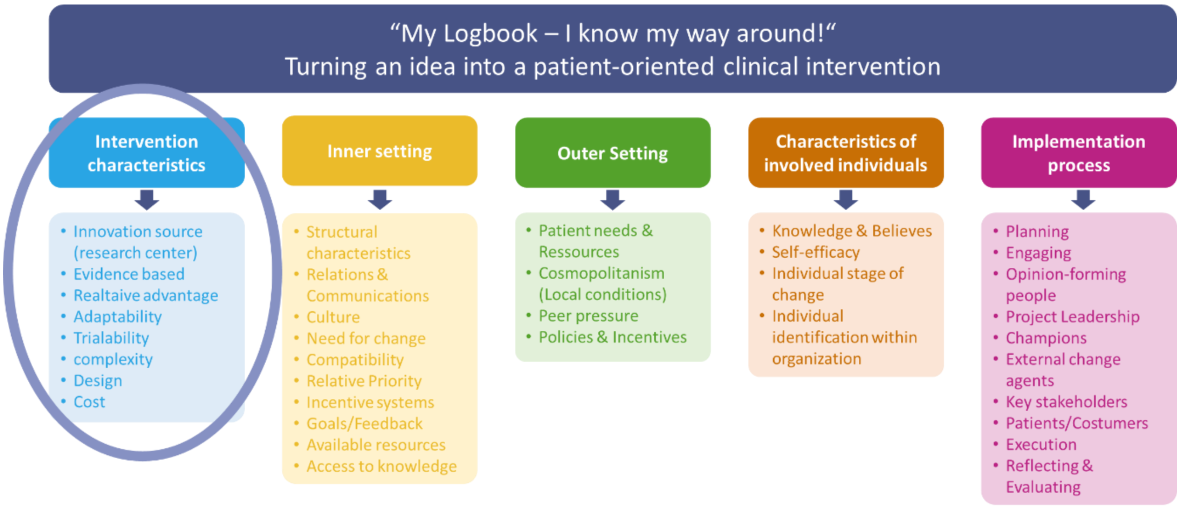

CFIR Subcategories	Explanation
Intervention source	Perception of key stakeholders about whether the intervention is externally or internally developed.
Evidence strength & quality	Stakeholders' perceptions of the quality and validity of evidence supporting the belief that the intervention will have desired outcomes.
Relative advantage	Stakeholders' perception of the advantage of implementing the intervention versus an alternative solution.
Adaptability	The degree to which an intervention can be adapted, tailored, refined, or reinvented to meet local needs.
Trialability	Stakeholders' perception if an intervention will be piloted prior to full-scale implementation.
Complexity	Perceived difficulty of implementation, reflected by duration, scope, radicalness, disruptiveness, centrality, and intricacy and number of steps required to implement.
Design quality & packaging	Perceived excellence in how the intervention is bundled, presented, and assembled.
Cost	Costs of the intervention and costs associated with implementing that intervention including investment, supply, and opportunity costs.

**The institute or the persons associated with it (Medical University of Vienna in cooperation with the PSAPOH) have the necessary competence for the development of the tool "My Logbook" (source of the intervention)**			
• Yes	• Partly	• No	• I cannot judge
**Comments, rationale, questions, suggested solutions:**			
(Open response)			

**The S3 guideline on mental health care is a valid and evidence-based foundation for "My Logbook" (strength and quality of evidence)**			
• Yes	• Partly	• No	• I cannot judge
**Comments, rationale, questions, suggested solutions:**			
(Open response)			

The tool "My Logbook" contributes to the implementation of the S3 guideline on psychosocial care in everyday clinical practice and to the recommendations from it reaching the patient (strength and quality of evidence).			
• Yes	• Partly	• No	• I cannot judge
**Comments, rationale, questions, suggested solutions:**			
(Open response)			

The tool "My Logbook" (as a combined tool for treatment and research) adds value, compared to existing methods, to get closer to the goal of guideline-based care (Relative advantage)			
• Yes	• Partly	• No	• I cannot judge

I find this particularly relevant/helpful in this context:
• Psychosocial expertise - in development and implementation
• Patient:inside-orientation - promotion of empowerment and action competence
• Standards with individuality - harmonization of daily clinical routines and maintaining diversity of methodology and individuality (objectivity)
• Evidence of effectiveness (therapy optimization study) - linking research and practice (showing together that it takes expertise, time resources and works what we do!
• Guideline-based - principles, goals & measures find immediate practical application
• Consensus-based - Interdisciplinary & International
• Combined tool of research and practice (linking research and practice)
• Making visible and strengthening the importance of psychosocial care and research (for patients and in the interdisciplinary team)
• To make visible possible gaps in care (e.g., exchange in the interdisciplinary team).
• Other comments/justifications/questions (I experienced this as helpful, ideas, suggestions for solutions, opportunities, advantages): ____________

The tool "My Logbook" addresses children/adolescents with an oncological or hematological disease between 6 and 14 years and can be flexibly adapted for this age group in order to meet clinic-specific framework conditions as well as patient-specific needs (adaptability).			
• Yes	• Partly	• No	• I cannot judge
**I find this particularly relevant/helpful in this context:**			
• Focus on the child as patient (age-appropriate support)			
• Concrete visualized methods (complex processes become tangible)			
• Modular structure (possibility to choose specific topics)			
• Homework (transfer to everyday life as well as the possibility to work on or deepen selected tasks and exercises at home)			
• Low-threshold contact (easier to establish contact with a family (keyword: "Icebreaker"))			
• Fixed modules create liabilities (appointments can be enforced sooner, additional appointments are booked)			
• Process-oriented screening (visibility over the course of emotional well-being and information level)			
• Flexible use throughout the entire PSD (depending on the respective focus)			
• Design with high incentive character (colors, stickers, handicraft sheets, orientation aids)			
• Design oriented to the target group (font size, readability, ...)			
• Design presentation (packaging, print quality, ...)			
• Other comments/reasons/questions (I found this helpful, ideas, suggested solutions, opportunities, advantages): ___________________			

The tool "My Logbook" can be tried out in a pilot phase in order to be able to take into account clinic-specific framework conditions as well as patient-specific needs (trialability).			
• Yes	• Partly	• No	• I cannot judge
**I find this particularly relevant/helpful in this context:**			
• Trainings (workshop & training films)			
• Study coordination meetings (networking and sharing).			
• Log "helpline" support (phone & email - (networking and sharing))			

• Social media (Slack, Facebook - (networking and sharing))
• Conference papers, written information, etc.
• Newsletter
• Manual (instructions as well as sample answers in the manual support a quick deployment and support a practical handling)
• Basic supply with integrated logbook modules
• Topic booklets and logbook
• Log sheets for process-oriented screening (standardized clinical assessment and achieved psychosocial goals)
• Evaluation tools
• Dashboard (presentation of scientific results)
• Other comments/reasons/questions (I found this helpful, ideas, suggested solutions, opportunities, benefits.

**Despite its complexity in terms of content, structure, materials and choice of methods, the tool "My Logbook" is clear. It thus reflects the complexity of the psychosocial process and thus supports the practical implementation of the S3 guideline (complexity)**			
• Yes	• Partly	• No	• I cannot judge
**Comments, rationale, questions, suggested solutions:**			
(Open response)			

**The "My Logbook" tool has an appealing design that is inviting to both patients and PSDs (design, quality, packaging).**			
• Yes	• Partly	• No	• I cannot judge
**I find this particularly relevant/helpful in this context:**			
• Focus on the child as patient (age-appropriate support)			
• Concrete visualized methods (complex processes become tangible)			
• Modular structure (possibility to select specific topics)			
• Homework (transfer to everyday life as well as the possibility to work on and deepen selected tasks and exercises at home).			
• to work on or deepen selected tasks and exercises at home)			
• Low-threshold contact (easier to establish contact with a family (keyword: "Icebreaker"))			
• Fixed modules create liabilities (appointments can be enforced more easily, additional appointments can be			
• are booked)			
• Process-oriented screening (visibility over the course of emotional well-being and			
• information levels)			
• Flexible use throughout the entire PSD (depending on the respective focus)			
• Design with high incentive character (colors, stickers, handicraft sheets, orientation aids)			
• Design oriented to the target group (font size, readability, ...)			
• Design presentation (packaging, print quality, ...)			
• Other comments/reasons/questions (I found this helpful, ideas, suggested solutions, chances, advantages): ________			

**The costs for the implementation of the "My Logbook" tool (including investment, material and personnel costs) are**			
• Yes	• Partly	• No	• I cannot judge
**Comments, rationale, questions, suggested solutions:**			
(Open response)			

**With regard to the key features of the patient-oriented tool "My logbook - I know myself" - it needs in my opinion still:**
(Open response)

Page 7

Inner Setting

The inner attitude is the basis for whether the implementation of a new tool like "My Logbook" becomes possible. It includes our own attitude as psychosocial professionals in the German-speaking countries - within the PSAPOH network or within the institution where each of us works.

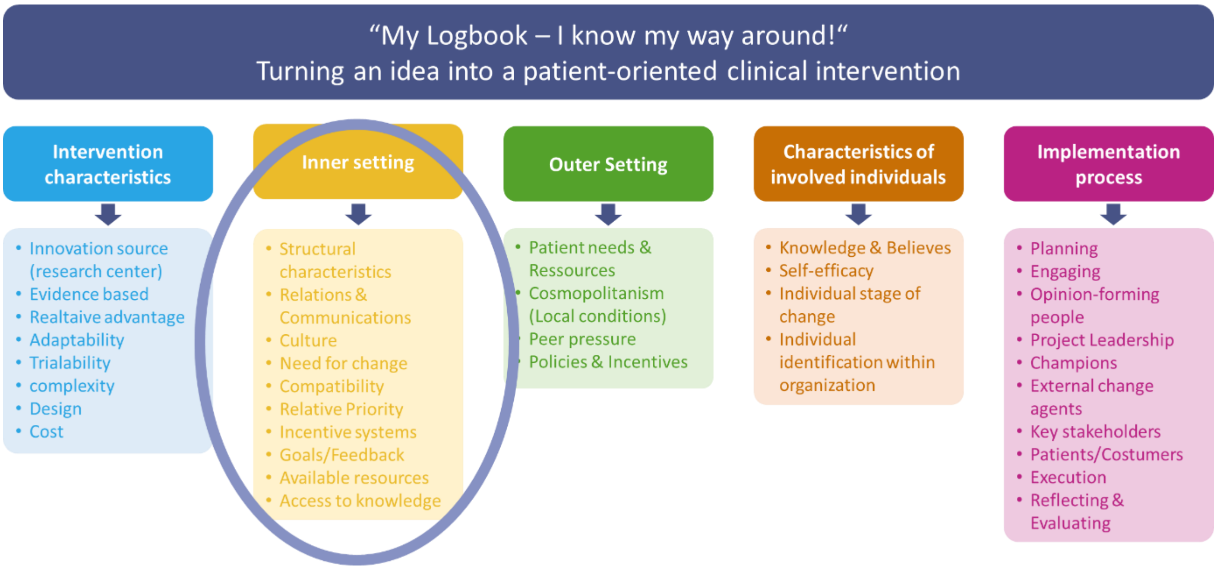

CFIR Subcategories	Explanation
Structural characteristics	Perception of influence of the social architecture, age, maturity, and size of an organization on the implementation of an intervention, and perception of necessary changes to the infrastructure to accommodate the intervention.
Relations & communication	The nature and quality of webs of social networks and the nature and quality of formal and informal communications within an organization.
Culture	Norms, values, and basic assumptions of a given organization.
Need for change	The degree to which stakeholders perceive the current situation as intolerable or needing change.
Compatibility	The degree of tangible fit between meaning and values attached to the intervention by involved individuals, how those align with individuals' own norms, values, and perceived risks and needs, and how the intervention fits with existing workflows and systems.
Relative priority	Individuals' shared perception of the importance of the implementation within the organization.
Incentive systems	Extrinsic incentives such as goal-sharing awards, performance reviews, promotions, and raises in salary and less tangible incentives such as increased stature or respect.
Goals/feedback	The degree to which goals are clearly communicated, acted upon, and fed back to staff and alignment of that feedback with goals.
Available resources	The level of resources dedicated for implementation and on-going operations including money, training, education, physical space, and time.
Access to knowledge	Ease of access to digestible information and knowledge about the intervention and how to incorporate it into work tasks.

**The framework conditions at the clinics/institutions enable the use of the "My Logbook" tool (Structural framework conditions)**			
• Yes	• Partly	• No	• I cannot judge
**I find this particularly challenging/obstructive in this context:**			
• Personal assessment of the importance, significance and explosiveness (of the project/topic)			
• Time resource			
• Personnel changes in the team (number of hours, sick leave, short contracts, lack of replacements during maternity/paternity leave, third-party funding...)			

• Study-specific organization (ethics, consent forms, data acquisition and transmission)
• Scientific know-how (ethics applications, consent forms, data protection, ...)
• Duration and scope of the individual topics
• Clinic-specific framework conditions (position at the clinic, ...)
• Pandemic and other daily crises
• Comments/reasons/questions/obstacles (what I experienced as challenging, ideas, proposed solutions, opportunities, advantages/disadvantages)

**The "My Log" tool is integrated via essential networks (PSAPOH or similar) and/or has formal and informal communication methods to convey the content (networks and communication)**			
• Yes	• Partly	• No	• I cannot judge
**I feel this is particularly relevant/helpful in this context:**			
• Trainings (workshop & training films)			
• Study coordination meetings (networking and sharing).			
• Log "helpline" support (phone & email - (networking and sharing))			
• Social media (Slack, Facebook - (networking and sharing))			
• Conference papers, written information, etc.			
• Newsletter			
• Manual (instructions as well as sample answers in the manual support a quick deployment and support a practical handling)			
• Basic supply with integrated logbook modules			
• Topic booklets and logbook			
• Log sheets for process-oriented screening (standardized clinical assessment and achieved psychosocial goals)			
• Evaluation tools			
• Dashboard (presentation of scientific results)			
• Notes/reasons/questions (I found this helpful, ideas, suggested solutions, opportunities, benefits): _____________			

**My Logbook tool is based on cultural norms, values and basic assumptions that support its use (culture)**			
• Yes	• Partly	• No	• I cannot judge
**I feel this is particularly relevant/helpful in this context:**			
• Psychosocial expertise - in development and implementation			
• Patient:inside-orientation - promotion of empowerment and action competence			
• Standards with individuality - harmonization of clinical daily routines and maintaining diversity of methodology and individuality (objectivity)			
• Evidence of effectiveness (therapy optimization study) - linking research and practice (showing together that it takes expertise, time resources and works what we do!			
• Guideline-based - principles, goals & measures find immediate practical application			
• Consensus-based - Interdisciplinary & International			
• Combined tool of research and practice (linking research and practice)			
• Making visible and strengthening the importance of psychosocial care and research (for patients and in the interdisciplinary team)			
• To make visible possible gaps in care (e.g., exchange in the interdisciplinary team).			
• Other comments/justifications/questions (I experienced this as helpful, ideas, suggestions for solutions, opportunities, advantages): __________			

**Within my institution, we have a receptiveness, willingness to change, and expectation that using a tool like "My Logbook" will be rewarded/supported/expected (implementation climate)**			
• Yes	• Partly	• No	• I cannot judge
**I find this particularly challenging/obstructive in this context:**			
• Personal assessment of the importance, significance and explosiveness (of the project/topic)			
• Time resource			
• Staffing changes in the team (number of hours, sick leave, short contracts, lack of			
• during maternity/paternity leave, third-party funding...)			

• Study-specific organization (ethics, consent forms, data acquisition and transmission)			
• Scientific know-how (ethics applications, consent forms, data protection, ...)			
• Duration and scope of the individual topics			
• Clinic-specific framework conditions (position at the clinic, ...)			
• Pandemic and other daily crises			
• Other comments, justifications, questions, proposed solutions: ____________			
**An improvement of the current situation of psychosocial care (standards, extent, importance, ...) is necessary and can be supported by the tool "My Logbook" (need for change)**			
• Yes	• Partly	• No	• I cannot judge
**The tool "My Logbook" is compatible with state of the art working methods, meaning and values of psychosocial care, complies with PSD requirements and/or contributes to quality assurance of activities (compatibility)**			
• Yes	• Partly	• No	• I cannot judge
**The tool "My logbook" has advantages compared to other methods/activities/interventions (relative priority)**			
• Yes	• Partly	• No	• I cannot judge
**Tangible incentives exist (e.g., continuing education credits, intervision confirmations of study coordination meetings, goal setting, service delivery) for using the "My Logbook" tool (organizational incentives/rewards)**			
• Yes	• Partly	• No	• I cannot judge
**Fewer tangible incentives exist (e.g., prestige, respect, recognition, visibility of mental health care) for using the My Logbook tool (Organizational Incentives/Rewards)**			
• Yes	• Partly	• No	• I cannot judge
**Goals (or the achievement of them) of the "My Logbook" tool are clearly communicated and implemented (goal/feedback)**			
• Yes	• Partly	• No	• I cannot judge
**The working atmosphere and the communication in the Logbook Experts group is appreciative and allows an involvement in all development stages of the tool "My Logbook" of all interested colleagues in the German-speaking area (conception and development, testing at the clinics) (learning climate)**			
• Yes	• Partly	• No	• I cannot judge

**There are tangible and immediate aspects (e.g. participation in development, availability of materials) that contribute to the readiness and commitment of mental health professionals to use the tool "My Logbook" (readiness for implementation)**			
• Yes	• Partly	• No	• I cannot judge
**I find this particularly challenging/obstructive in this context:**			
• Trainings (workshop & training films)			
• Study coordination meetings (networking and sharing).			
• Log "helpline" support (phone & email - (networking and sharing))			
• Social media (Slack, Facebook - (networking and sharing))			
• Conference papers, written information, etc.			
• Newsletter			
• Manual (instructions as well as sample answers in the manual support a quick deployment and support a practical handling)			
• Basic supply with integrated logbook modules			
• Topic booklets and logbook			
• Log sheets for process-oriented screening (standardized clinical assessment and achieved psychosocial goals)			
• Evaluation tools			
• Dashboard (presentation of scientific results)			
• Other comments/justifications/questions (I found this helpful, ideas, suggested solutions, opportunities, benefits)			
**Leaders in my institution (hospital directors, senior physicians, ...) were involved/informed so that the tool "My Logbook" is applied (Leadership engagement)**			
• Yes	• Partly	• No	• I cannot judge
**Sufficient resources (e.g. funding, time, premises) are available at my institution for the implementation of guideline-based tools such as "My Logbook" (Available resources)**			

• Yes	• Partly	• No	• I cannot judge
**Understandable information about the tool "My Logbook" or instructions for its use (e.g. manual, ...) are provided. (Access to knowledge & information)**			
• Yes	• Partly	• No	• I cannot judge

**In order for an implementation of the patient-oriented tool "My logbook - I know my way around!" to be possible, in my opinion, with regard to the "inner attitude", ...**
(Open response)

Page 8

Outer setting

The external framework comprises the addressed target group (children between 6-14 years of age with an oncological or oncological or hematological disease) as well as the general conditions in the health care system.

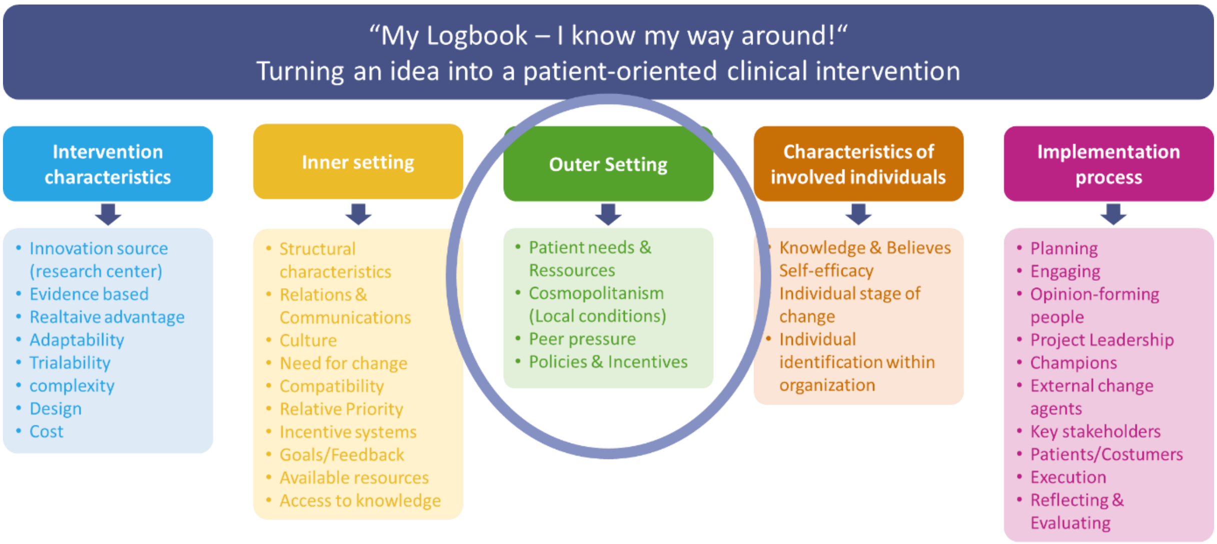

CFIR Subcategories	Explanation
Patient needs & resources	The extent to which patient needs, as well as barriers and facilitators to meet those needs are accurately known and prioritized by the organization.
Cosmopolitanism	The degree to which an organization is networked with other external organizations.
Peer pressure	Perception of pressure to implement an intervention; typically because most or other key peer or competing organizations have already implemented or in a bid for a competitive edge.
External policies & incentives	A broad construct that includes external strategies to spread interventions including policy and regulations, external mandates, recommendations and guidelines, pay-for-performance, collaboratives, and public or benchmark reporting.

**The tool "My Logbook" addresses the needs of the patient (stresses) and picks up support possibilities (resources). (Patient needs & resources)**			
• Yes	• Partly	• No	• I cannot judge
**Comments, rationale, questions, suggested solutions:**			
(Open response)			

**There is a regular networking between the institute or the persons connected with it (Medical University of Vienna in cooperation with PSAPOH) and the professional groups as well as patient organizations of the D-A-CH region. (Cosmopolitanism)**			
• Yes	• Partly	• No	• I cannot judge
**I feel this is particularly relevant/helpful in this context:**			
• Trainings (workshop & training films)			
• Study coordination meetings (networking and exchange)			
• Log "helpline" support (phone & email - (networking and sharing))			
• Social media (Slack, Facebook - (networking and sharing))			
• Conference papers, written information, etc.			
• Newsletter			
• Manual (instructions as well as sample answers in the manual support a quick deployment and support a practical handling)			
• Basic supply with integrated logbook modules			

• Topic booklets and logbook
• Log sheets for process-oriented screening (standardized clinical assessment and achieved psychosocial goals)
• Evaluation tools
• Dashboard (presentation of scientific results
**Comments, rationale, questions, suggested solutions:**
(Open response)

**The "My Logbook" tool is "standard of care" at my institutions or an integral part of routine care (group affiliation)**			
• Yes	• Partly	• No	• I cannot judge
**Comments, rationale, questions, suggested solutions:**			
(Open response)			

**Standards, specifications, policy frameworks, recommendations or guidelines, pay-for-performance, integration into social insurance benefits, collaborations, or public or benchmark reports (policy & incentives) exist for the implementation of the "My Logbook" tool.**			
• Yes	• Partly	• No	• I cannot judge
**Comments, rationale, questions, suggested solutions:**			
(Open response)			

**In order for an implementation of the patient:inside oriented tool "My logbook - I know my way around!" to be possible, I think that with regard to the "external framework" it still needs, ...<br>(wishes, suggestions, ideas, proposed solutions, ...)**
(Open response)

Involvement

Organizations are composed of individuals. Attitudes and intervention constructs are ultimately grounded in the actions and behaviors of individuals (A broad construct that also includes other personal characteristics such as tolerance for ambiguity, intellectual ability, motivation, values, competence, capacity, and learning style). In the assessment, the focus is on you or the PSD in which you work or the patient group and their family.

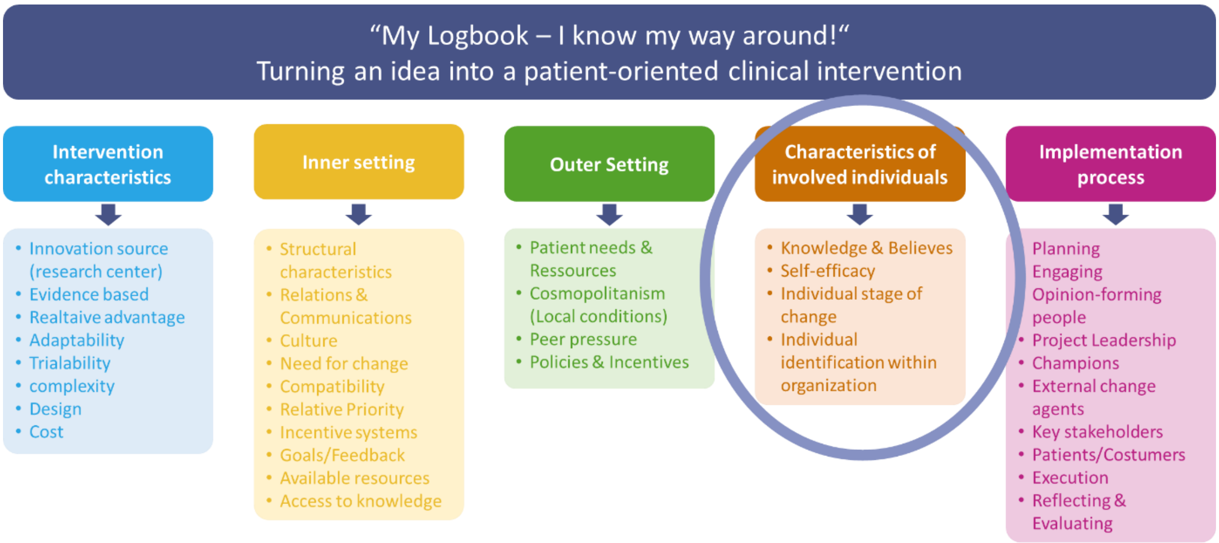

CFIR Subcategories	Explanation
Knowledge & beliefs about the intervention	Individuals' attitudes toward and value placed on the intervention as well as familiarity with facts, truths, and principles related to the intervention.
Self-efficacy	Individual belief in their own capabilities to execute courses of action to achieve implementation goals.
Individual stage of change	Characterization of the phase an individual is in, as he or she progresses toward skilled, enthusiastic, and sustained use of the intervention.
Individual identification with organization	A broad construct related to how individuals perceive the organization and their relationship and degree of commitment with that organization.
Other personal attributes	A broad construct to include other personal traits such as tolerance of ambiguity, intellectual ability, motivation, values, competence, capacity, and learning style.

**I show a positive attitude towards "My Logbook", experience it as valuable and feel addressed by its presentation and principles (knowledge & beliefs)**			
• Yes	• Partly	• No	• I cannot judge

**The patients and their families show a positive attitude towards "My Logbook", experience it as valuable and feel that the presentation and principles address their needs. The processing is a pleasure. (Knowledge & Beliefs)**			
• Yes	• Partly	• No	• I cannot judge
**Comments, rationale, questions, suggested solutions:**			
(Open response)			

**I have confidence in our abilities to implement "My Logbook" or related actions to achieve implementation goals (self-confidence/self-efficacy).**			
• Yes	• Partly	• No	• I cannot judge

**Patients and their families have confidence in their abilities to achieve the addressed examination and treatment goals because of "My Logbook". (Self-confidence/self-efficacy)**			
• Yes	• Partly	• No	• I cannot judge
**Comments, rationale, questions, suggested solutions:**			
(Open response)			

**I am able or convinced to use "My Logbook" in a sustainable way (Individual stage of change)**			
• Yes	• Partly	• No	• I cannot judge

**The patients and their families are able or convinced to use "My Logbook" in a sustainable way. (Individual stage of change)**			
• Yes	• Partly	• No	• I cannot judge
**Comments, rationale, questions, suggested solutions:**			
(Open response)			

**I show a high level of commitment through my participation due to the guidance of the study center (Med Uni Vienna) (Individual identification with "My Logbook")**			
• Yes	• Partly	• No	• I cannot judge

**The patients and their families show a willingness to cooperate with "My Logbook" due to the guidance of the PSD in charge of the case (Individual identification with "My Logbook")**			
• Yes	• Partly	• No	• I cannot judge
**Comments, rationale, questions, suggested solutions:**			
(Open response)			

**In order for an implementation of the patient:inside oriented tool "My logbook - I know my way around!" to be possible, it still needs, in my opinion, with regard to the "Involved persons", ...<br>(Free field for wishes, suggestions, ideas, solution suggestions, ...)**
(Open response)

Page 10

Implementation process

The implementation process incorporates theories from quality management, involvement, complexity theory, organizational learning, etc.

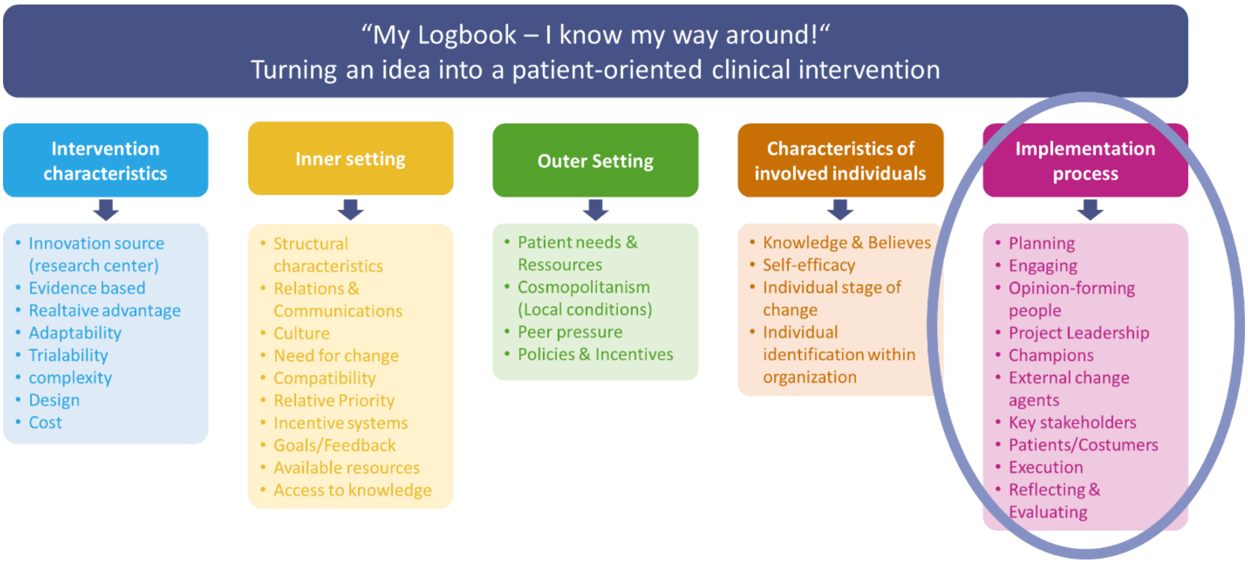

CFIR Subcategories	Explanation
Planning	The degree to which a scheme or method of behavior and tasks for implementing an intervention are developed in advance and the quality of those schemes or methods.
Engaging	Attracting and involving appropriate individuals in the implementation and use of the intervention through a combined strategy of social marketing, education, role modeling, training, and other similar activities.
Opinion-forming people	Individuals in an organization who have formal or informal influence on the attitudes and beliefs of their colleagues with respect to implementing the intervention.
Project leadership	Individuals from within the organization who have been formally appointed with responsibility for implementing an intervention as coordinator, project manager, team leader, or other similar role.
Champions	Individuals who dedicate themselves to supporting, marketing, and 'driving through' an implementation, overcoming indifference or resistance that the intervention may provoke in an organization.
External change agents	Individuals who are affiliated with an outside entity who formally influence or facilitate intervention decisions in a desirable direction.
Key stakeholders	Individuals who are needed to be on board with the intervention.
Patients/customers	Individuals who will receive the intervention.
Executing	Carrying out or accomplishing the implementation according to plan.
Reflecting & evaluating	Quantitative and qualitative feedback about the progress and quality of implementation accompanied with regular personal and team debriefing about progress and experience.

**The tool "My Logbook" has clear and quality action instructions for the application and implementation (planning)**			
• Yes	• Partly	• No	• I cannot judge
**I find this particularly relevant/helpful in this context:**			
• Psychosocial expertise - in development and implementation			
• Patient -orientation - promotion of empowerment and action competence			
• Standards with individuality - harmonization of daily clinical routines and maintenance of the			
• diversity of methodology and individuality (objectivity)			

• Evidence of effectiveness (therapy optimization study) - linking research and practice (showing together that it takes expertise, time resources and works what we do!
• Guideline-based - principles, goals & measures find immediate practical application
• Consensus-based - Interdisciplinary & International
• Combined tool of research and practice (linking research and practice)
• Making visible and strengthening the importance of psychosocial care and research (for
• patients and in the interdisciplinary team)
• To make visible possible gaps in care (e.g., exchange in the interdisciplinary team).
• Other comments, justifications, questions, suggestions for solutions: _______

**In the development of the "My Logbook" tool, appropriate individuals were recruited or involved in the implementation and use of the intervention through a combined strategy of promotion, outreach, education, and other similar activities (Engaging)**			
• Yes	• Partly	• No	• I cannot judge
**I feel this is particularly relevant/helpful in this context:**			
• Focus on the child as patient (age-appropriate support)			
• Concrete visualized methods (complex processes become tangible)			
• Modular structure (possibility to choose specific topics)			
• Homework (transfer to everyday life as well as the possibility to work on or deepen selected tasks and exercises at home)			
• Low-threshold contact (easier to establish contact with a family (keyword: "Icebreaker"))			
• Fixed modules create liabilities (appointments can be enforced sooner, additional appointments are booked)			
• Process-oriented screening (visibility over the course of emotional well-being and information level)			
• Flexible use throughout the entire PSD (depending on the respective focus)			
• Design with high incentive character (colors, stickers, handicraft sheets, orientation aids)			
• Design oriented to the target group (font size, readability, ...)			
• Design presentation (packaging, print quality, ...)			
• Training (workshop & training films)			
• Study coordination meetings (networking and exchange)			
• Log "helpline" support (phone & email - (networking and sharing))			
• Social media (Slack, Facebook - (networking and sharing))			
• Conference papers, written information, etc.			
• Newsletter			
• Manual (instructions as well as sample answers in the manual support a quick deployment and support a practical handling)			
• Basic supply with integrated logbook modules			
• Topic booklets and logbook			
• Log sheets for process-oriented screening (standardized clinical assessment and achieved psychosocial goals)			
• Evaluation tools			
• Dashboard (presentation of scientific results)			
• Other comments, rationales, questions, suggested solutions: ______			
**Psychosocial experts, interdisciplinary colleagues and stakeholders (patients & families) are involved in the development of the tool "My Logbook" and support the project. (Opinion leaders (people who formally or informally influence the attitudes and beliefs of their colleagues regarding the implementation of the intervention))**			
• Yes	• Partly	• No	• I cannot judge
**Project manager/lead, study coordinators who have responsibility for implementing "My Logbook" have been formally appointed and are recognized within the PSAPOH network and their own institution, respectively. (Project Manager)**			
• Yes	• Partly	• No	• I cannot judge
**Individuals (in role model/leader functions) have been involved in the implementation and support "My Logbook" (Champions)**			
• Yes	• Partly	• No	• I cannot judge

**Individuals from an external entity (e.g., PSAPOH, institutional sponsors, social service agencies) have been involved and support the project to facilitate formal decision-making and use of "My Logbook" (External Change Agent)**			
• Yes	• Partly	• No	• I cannot judge
**There are sufficient and effective measures in place to engage and involve key stakeholders in the implementation or use of "My Logbook" (e.g., through conference presentations, newsletters, publications, training/educational film). (Key stakeholder positions)**			
• Yes	• Partly	• No	• I cannot judge
**There are sufficiently effective strategies to attract and involve patients in the implementation or use of "My Logbook" (e.g. reports in PTA magazines, flyers, information through the PSD, newsletters). (Patients)**			
• Yes	• Partly	• No	• I cannot judge

**The measures for the implementation of the "My Logbook" tool are implemented as planned. (Implementation)**			
• Yes	• Partly	• No	• I cannot judge
**I find this particularly challenging/disruptive in this context:**			
• Personal assessment of the importance, significance and explosiveness (of the project/topic)			
• Time resource			
• Staffing changes in the team (number of hours, sick leave, short contracts, lack of			
• during maternity/paternity leave, third-party funding...)			
• Study-specific organization (ethics, consent forms, data acquisition and transmission)			
• Scientific know-how (ethics applications, consent forms, data protection, ...)			
• Duration and scope of the individual topics			
• Clinic-specific framework conditions (position at the clinic, ...)			
• Pandemic and other daily crises			
• Other comments, rationales, questions, proposed solutions: _______			

**There is quantitative (dashboard, publications) and qualitative feedback (newsletters, conferences, student coordinator meetings, regular face-to-face and team debriefings on progress and lessons learned) on the progress and quality of the implementation/application of "My Logbook" (Reflect and Evaluate)**			
• Yes	• Partly	• No	• I cannot judge
**I feel this is particularly relevant/helpful in this context:**			
• Trainings (workshop & training films)			
• Study coordination meetings (networking and sharing).			
• Log "helpline" support (phone & email - (networking and sharing))			
• Social media (Slack, Facebook - (networking and sharing))			
• Conference papers, written information, etc.			
• Newsletter			
• Manual (instructions as well as sample answers in the manual support a quick deployment and support a practical handling)			
• Basic supply with integrated logbook modules			
• Topic booklets and logbook			
• Log sheets for process-oriented screening (standardized clinical assessment and achieved psychosocial goals)			
• Evaluation tools			
• Dashboard (presentation of scientific results)			
• Other comments, justifications, questions, proposed solutions			

**"In order for an implementation of the patient:inside oriented tool "My logbook - I know my way around!" to be possible, I think it still needs, with regard to the "implementation process", ... (Free field for wishes, suggestions, ideas, proposed solutions, ...)"**
(Open response)
